# Neonatal screening for congenital hypothyroidism: primary thryotropin screening: comparison of false positive rate using radioimmunossay (RIA) Vs fluorometric assay (FIA)

**DOI:** 10.1186/1687-9856-2013-S1-P151

**Published:** 2013-10-03

**Authors:** Diet S Rustama, Elly Rosilawati, Vidi Permatagalih

**Affiliations:** 1 Refference Centre of Neonatal Screening Faculty of Medicine, Padjadjaran University, Hasan Sadikin General Hospital, Bandung, West Java, Indonesia

**Keywords:** TSH primary screening, congenital hypothyroidism, false positive rate

## Background

In Indonesia one of the challenges in implementing neonatal screening for congenital hypothyroidism (CH) is early discharge of infants, therefore we could not avoid to take the specimens between 24 to 48 hours of age, resulted in high false-positive rate. Since the begining of the neonatal screening program, radioimmunoassay (RIA) was used for primary TSH screening. Fluoroimmunoassay (FIA) are expected to be more sensitive method.

## Objective

To compare the results of TSH measurements, using RIA and FIA in efforts to minimize false positive rate.

## Methods

Blood-spotted filter paper specimens, obtained by heel prick for primary TSH screening were collected from neonates born in 12 hospitals in Bandung. Low birth weight infants, sick neonates, and specimens collected less than 24 ours of age were excluded. In 2008, RIA (Coat-A-Count, Neonatal TSH IRMA), and in 2009 FIA (DELFIA Neonatal hTSH) was used. The cutoff value of TSH was 20mU/L. We calculated the false positive results on specimens obtained within 24-48 hours after birth and those taken after 48 hours. The false positive rate compared within two assays.

## Results

Specimens from 7915 babies were measured by RIA, 53.46 % were collected before 48 hours of age, 36.18% between 24 to 48 hours, and 7684 babies were measured by FIA, 61.17% were collected before 48 hours of age and 39.43% between 24 to 48 hours. Healthy, fullterm babies screened by RIA at age after 24 hours were 6525 with 0.86% recall rate and 0.83% false positive rate. Six thousands and thirteen specimens were measured by FIA, with 0.17% recall rate and 0.13% false positive rate, significantly lower than RIA (p <0.05). Using FIA recall rate and false positive rate on specimen collected between 24 to 48 hours are 0.16% and 0.16%, while on specimen collected after 48 hours are 0.17% and 0.10%, not significantly different (p=0.07).

## Conclusions

Primary TSH screening for congenital hypothyroidism using FIA is more sensitive RIA. Lower recall rate as well as false positive rate decreased the burden of tracking the infants for confirmatory, therefore switching the test to FIA is appropriately reasonable.

